# Sex chromosome differentiation in *Humulus japonicus* Siebold & Zuccarini, 1846 (Cannabaceae) revealed by fluorescence *in situ* hybridization of subtelomeric repeat

**DOI:** 10.3897/CompCytogen.v6i3.3261

**Published:** 2012-07-10

**Authors:** Oleg S. Alexandrov, Mikhail G. Divashuk, Nikolay A. Yakovin, Gennady I. Karlov

**Affiliations:** 1 Centre for Molecular Biotechnology, Russian State Agrarian University – Moscow Timiryazev Agricultural Academy, Moscow 127550, Timiryazevskaya Street, 49, Russia

**Keywords:** *Humulus japonicus*, sex chromosomes, sex determination in plants, subtelomeric repeat, fluorescence *in situ* hybridization

## Abstract

*Humulus japonicus* Siebold et Zucc (Japanese hop) is a dioecious species of the family Cannabaceae. The chromosome number is 2n = 16 = 14 + XX for females and 2n = 17 = 14 + XY1Y2 for male. To date, no fluorescence *in situ* hybridization (FISH) markers have been established for the identification of *Humulus japonicus* sex chromosomes. In this paper, we report a method for the mitotic and meiotic sex chromosome differentiation in *Humulus japonicus* by FISH for HJSR, a high copy subtelomeric repeat. The signal is present in the subtelomeric region of one arm of the X chromosome. We demonstrate that males have two Y chromosomes that differ in FISH signal with the HJSR probe. Indeed, the HJSR probe hybridizes to a subtelomeric region on both arms of chromosome Y1 but not of chromosome Y2. The orientation and position of pseudoautosomal regions (PAR1 and PAR2) were also determined.

## Introduction

*Humulus japonicus* Siebold & Zuccarini, 1846 (Japanese hop) is a dioecious, climbing and annual species of the family Cannabaceae. The chromosome number is 2n = 16 = 14 + XX for females and 2n = 17 = 14 + XY1Y2 for males. The sex of *Humulus japonicus* is determined by the ratio of X chromosomes and autosomes sets (A); a X:A ratio of 1.0 results in a female and a ratio of 0.5 results in a male (the Y chromosomes are dispensable) (Bridges 1921, Parker and Clark 1991, Shephard et al. 2000, Ming et al. 2007, Ming et al. 2011). The Y chromosomes in *Humulus japonicus* are markedly heterochromatic (Shephard et al. 2000). The multiple sex chromosome system (XX/XY1Y2) is similar to that of *Rumex acetosa* Linnaeus 1753, a model species in studies of sex determination and sex chromosome organisation in plants (Ruiz Rejón et al. 1994).

The closest relative of *Humulus japonicus* is the common hop *Humulus lupulus* Linnaeus, 1753.* H. lupulus* has the same sex determination system as in Japanese hop (X/A) but it differs in chromosome number (2n = 20 in both female and male plants) and in sex chromosome systems (XX/XY) (Winge 1929, Ono 1955, Shephard et al. 2000). Molecular phylogenetic analyses of cpDNA and nuclear rDNA coding regions in *Humulus lupulus* and *Humulus japonicus* haverevealed the high similarity of these two species. The time of divergence between these two species was estimated to be 6.38 million years ago (Murakami et al. 2006).

Due to the great economic importance of *Humulus lupulus*, molecular methods to assess genetic variability and genome organisation have been developed for this species. To understand sex chromosome evolution and organisation in plants, sex-linked genetic and cytogenetic markers are required. Male-specific DNA markers have been identified in *Humulus lupulus* (Polley et al. 1997, Seefelder et al. 2000, Danilova and Karlov 2006, Jakse et al. 2008) and recently in *Humulus japonicus* (Alexandrov et al. 2011).

Cytogenetic markers of *Humulus lupulus* sex chromosomes were established by application of C-banding/DAPI (Karlov et al. 2003) and FISH with a subtelomeric repeat (HSR1) as a probe (Divashuk et al. 2011). Cytogenetic analysis of *Humulus japonicus* has been limited, and little is known about the molecular cytogenetic organisation of the *Humulus japonicus* sex chromosomes. After conventional staining, autosomes and sex chromosomes cannot be morphologically distinguished from each other. Recently, molecular characterisation of the *Humulus japonicus* karyotype was completed by Kim et al. (2008) and Grabowska-Joachimiak et al. (2011). Using telomere repeats, 5S and 45S rDNA probes and C-banding/DAPI staining, a fluorescent karyotype was constructed. The latter study demonstrated that sex chromosomes of *Humulus japonicus* display unique DAPI banding patterns. The X chromosome possesses only one brightly stained AT-rich terminal segment, while Y1 has 2 such segments, and Y2 have no DAPI positive signal. This distribution of signal and the large size of the sex chromosomes allowed the authors to distinguish them from the autosomes and each other (Grabowska-Joachimiak et al. 2011).

A trivalent formation comprising Y1-X-Y2 associated with terminal chiasmata has been observed during meiosis in *Humulus japonicus* (Shephard et al. 2000, Kim et al. 2008). However, these observations were made without the benefit of cytogenetic markers for sex chromosomes, and nothing is known about their orientation in the trivalent formation.

To date, no FISH markers have been established for the identification of *Humulus japonicus* sex chromosomes. In this paper, we report a method for sex chromosome differentiation in *Humulus japonicus* by FISH with the subtelomeric repeat HJSR on mitotic and meiotic chromosomes.

## Material and methods

Male and female plants of *Humulus japonicus* raised from seeds of cv. Samuray (“Gavrish seeds”, Moscow, Russia) and seed lot № 4 (“Flos”, Moscow, Russia) were used in this study.

Total genomic DNA was isolated from young leaf material using the CTAB method (Rogers and Bendich 1985). To isolate the *Humulus japonicus* subtelomeric repeat HJSR, the DNA was digested by various restriction enzymes (*Alu*I, *Dra*I, *Eco*RI, *Hin*6I, *Hinc*II, *Hind*III, *Kpn*I, *Not*I, *Pst*I, *Xmi*I, *Bc*I, *Hae*III, *Vha*464I, *Bam*HI, *Nco*I, *Taq*I). The bands obtained after gel electrophoresis were carefully cut out from the gel, and the DNA was eluted with the QIAquick Gel Extraction Kit. The cloning of the eluted DNA was performed with the pUC 19 vector. Nucleotide sequences were determined using an ABI 3130 XL (Applied Biosystems) after sequencing reactions with a Big Dye Terminator v 1.1. Cycle Sequencing Kit (Applied Biosystems). BLAST analysis was performed according to standard procedures.

Mitotic metaphase chromosomes were prepared from fast growing root tip meristems collected from plants. They were pre-treated in 0.01 % α-bromonaphtalene at 4°C for 24 h and fixed in 3:1 (v/v) 96% ethanol: glacial acetic acid at room temperature for 1 h. For preparation of the microscopic slides, the root tips were rinsed in running water for 1 h and in distilled water three times and then were incubated in a 10 mM citrate buffer (pH 4.9) containing 0.4 % cellulase Onozuka R10 (Serva, Germany) and 0.2 % pectolyase Y-23 at 37°C for 3 h. Afterwards, the macerated root tips were spread by dissecting the tissue in 60 % acetic acid and by squashing it under a coverslip.

For meiotic chromosome preparations, the young anthers about 3-5 mm long at metaphase I were collected and fixed directly in acetic-ethanol (1:3) for 1 h, rinsed in water and then incubated for 2–3 hours in pectolytic enzymes containing 0.8 % cellulase Onozuka R10 (Serva, Germany) and 0.4 % pectolyase Y-23 in a 10 mM citrate buffer (pH 4.9). After two washes in distilled water, the anthers were carefully transferred onto grease-free slides, and the pollen mother cells were dissected out of the anther into a 1 μl droplet of water. Then, 5 μl of 60 % acetic acid was added, and the pollen mother cells were left for 2–3 minutes until the cytoplasm became sufficiently clear. The cells were then squashed under a coverslip.

For fluorescence *in situ* hybridization (FISH), the plasmid with the *Humulus japonicus* HJSR subtelomeric repeat DNA was labelled with dioxigenin-11-dUTP. The 1 μg sample of the purified DNA was labelled by nick translation according to the manufacturer’s protocol (Roche Diagnostics Gmbh, Germany). The chromosome and probe denaturation as well as hybridization and posthybridization washes were performed as described by Karlov et al. (2003). The chromosome preparations were counterstained with 5 μg/ml propidium iodide and mounted in Vectashield (Vector Laboratories, UK).

For detection of *Arabidopsis*-type telomere repeat in *Humulus japonicus* chromosomes sequential FISH was applied. Cover glasses were carefully removed after by washing for 1 h with 0.2 % Tween 20. Probe DNA was dissociated from the chromosomes with 70 % formamide in 2×SSC for 5 min. Slides were the dehydrated for 3 min each of 70, 90 and 100 % (v/v) ethanol, and air-dried. A new hybridization mix was added to the slides. The *Arabidopsis*-type telomere probe used was the deoxyribinucleotide oligomer (5’-CCCTAAA-3’)_3_ synthesised with a TAMRA label (ZAO “Syntol”, Moscow, Russia) at the 5’ end. The chromosome preparations were counterstained with DAPI.

The slides were observed under an AxioImager.M1 fluorescent microscope, photographed with a monochrome AxioCam MRm CCD camera, and visualised using Axiovision software (Carl Zeiss). In each experiment, at least 35 chromosome plates were analysed.

## Results

The isolated and cloned HJSR *Kpn*I-repeat was sequenced and found to be 380 bp in length (GU831573). No sex specific differences have been found between the sequences of male and female plants. The consensus sequence of 380 bp fragment is 63.4 % AT and does not possess any direct or inverted sequences of significant length. The BLAST analysis did not reveal any significant homology with sequences of other organisms.

**Figure 1.**
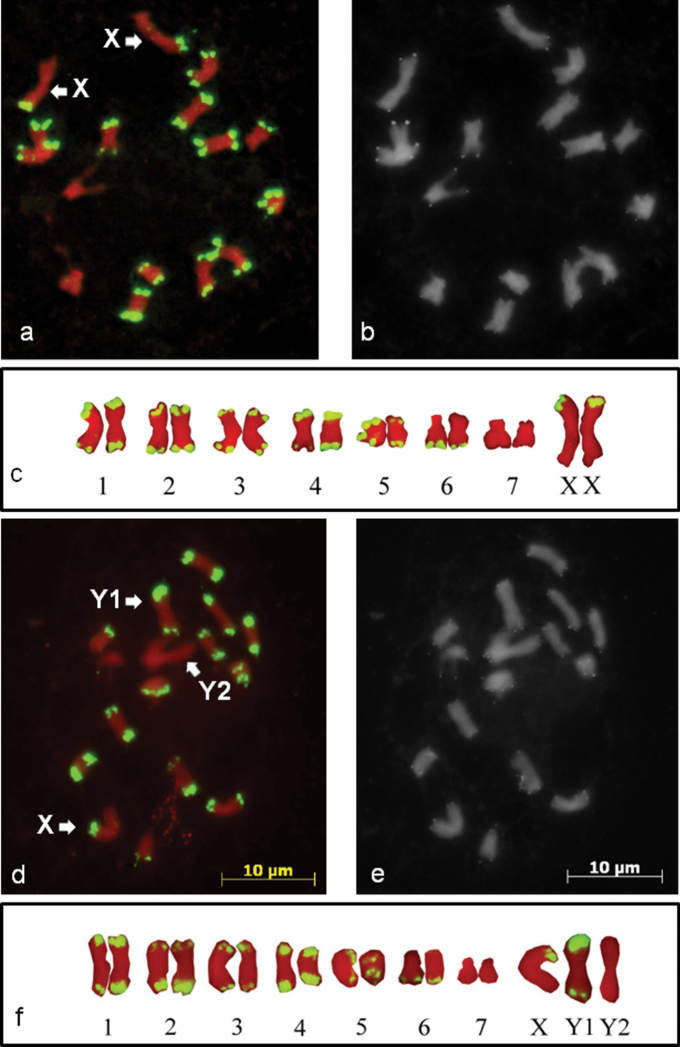
The mitotic chromosomes of *Humulus japonicus*. The chromosomes are counterstained by propidium iodide (red). The high copy HJSR subtelomeric repeat (green) is mapped to the female **(a)** and male **(d)** mitotic chromosomes of *Humulus japonicus* by FISH. The X, Y1 and Y2 chromosomes are marked by arrows. Sequential FISH with the *Arabidopsis*-type telomeric repeat on metaphase chromosomes of female (**b)** and male plants **(e).** The katyotypes of female **(c)** and male **(f)** plants.

The FISH signals observed with the HJSR probe were localised to subtelomeric regions of the chromosomes, and the signals were observed at one or both distal ends of each chromosome in both males and females. However, the signal was completely absent on one pair of autosomes from males and females and additionally on one of the three biggest chromosomes from males (Fig. 1). The FISH signal colocalised with the subtelomeric DAPI positive bands. No signal was detected from the interstitial regions of the chromosomes. The metaphase plates of the male and female plants were compared and revealed that the female metaphase plates carry two X chromosomes with the HJSR repeat signal on one of the arms (Fig. 1a, c). The male metaphase plates appeared to possess chromosome X with one signal, chromosome Y1 with signals on both arms and chromosome Y2 with no signal (Fig. 1d, f). FISH of the mitotic chromosomes of *Humulus japonicus* with a probe for an *Arabidopsis*-type telomere repeat showed signals on the all chromosome ends of both male and female plants (Fig 1b, e). The locations of the FISH signals from the telomeric probe were more distal from the centromere than those with the HJSR probe. No interstitial *Arabidopsis*-type telomere repeat signals were observed on the chromosomes.

**Figure 2.**
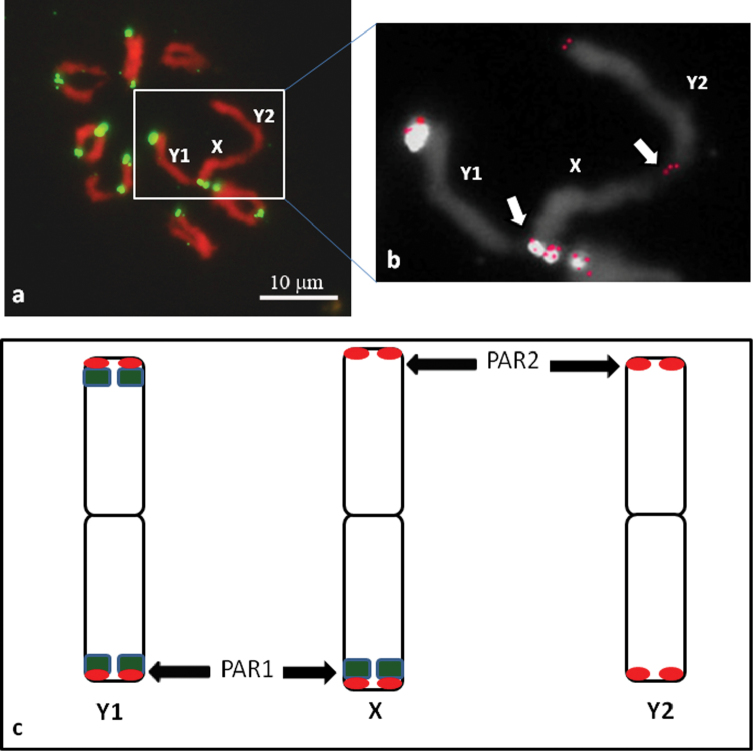
The meiotic chromosomes of *Humulus japonicus* at diakinesis with FISH signals for the HJSR repeat (green). The trivalent Y1-X-Y2 formation and chiasmata between the sex chromosomes can be clearly observed **(a).** The trivalent Y1-X-Y2 formation from **(a)** with combined signal of *Arabidopsis*-type telomeric repeat after sequential FISH (red) **(b)**. Schematic diagram of the *Humulus japonicus* X, Y1 and Y2 chromosomes **(c)** with the hybridization of the HJSR probe (green) and the *Arabidopsis*-type telomeric repeat probe (red). The pseudoautosomal regions (PAR1 and PAR2) are indicated by the arrows.

The results of the mitotic metaphase plate analyses are in agreement with the physical mapping of the HJSR to the meiotic chromosomes at diakinesis (Fig. 2). We identified the Y1-X-Y2 trivalent formation (Fig. 2a, b). The different ends of the X chromosome pair with different Y chromosomes. The Y1 chromosome, revealed HJSR FISH signals on both arms, pairs with arm of the X chromosome also carrying HJSR FISH signal. The Y2 chromosome has no HJSR FISH signal and pairs with the X chromosome arm that lacks a signal. This finding allows us to conclude that the pseudoautosomal regions (PAR1 and PAR2) are located at distal parts of both arms of the X chromosome and distally on one arm of each Y chromosome (Fig. 2c).

## Discussion

Most satellite DNAs are specific at the species or species subgroup levels (Kazama et al. 2003). Their presence and distribution reflect evolutionary events (Kubis et al. 1998, Koo et al. 2010). In this study, we isolated and described for the first time a new satellite DNA subtelomeric repeat, HJSR. This satellite DNA is localised at subtelomeric positions and colocalises with DAPI positive bands, except for the interstitial DAPI positive bands on chromosomes 3 and 7 discovered by Grabowska-Joachimiak et al. (2011). This signal pattern is in agreement with the DAPI staining, which detects AT-rich regions such as HJSR (63.4 %). The absence of this repeat in interstitial DAPI bands of chromosomes 3 and 7 indicates the presence of another type of AT-rich repeat. Interestingly, there are differences between the chromosomes with subtelomeric HJSR repeats on one or both arms, and the distribution of signal on the sex chromosomes is also different. These observations suggest the occurrence of chromosome reorganisation, implying that duplications or deletions may have occurred. In the common hop (*Humulus lupulus* L.), a *Kpn*I species specific subtelomeric repeat (HSR1) has also been cloned (Divashuk et al. 2011). The chromosome organisation of *Humulus lupulus* X chromosomes, as assessed by FISH for a subtelomeric repeat, was different from that of *Humulus japonicus*. Unlike *Humulus japonicus*, *Humulus lupulus* X chromosomes contain an interstitial HSR1 subtelomeric repeat site near the centromere. This difference reveals a karyotype reorganisation and sex chromosome evolution among these two closely related species. This also explains the different number and position of 5S and 45S rDNA loci on the autosomes of these two species (in *Humulus lupulus* 2 and 1, and in *Humulus japonicus* 1 and 2 loci, respectively) (Karlov et al. 2003, Kim et al. 2008, Grabowska-Joachimiak et al. 2011). Also, a higher amount of nuclear DNA has been found in *Humulus lupulus* (2C = 5.6 pg vs. 3.2 pg) (Grabowska-Joachimiak et al. 2006). The lower DNA content in *Humulus japonicus* may be due to the loss of subtelomeric repeats, as shown in our study for one pair of autosomal chromosomes and chromosome Y2. In contrast, all chromosomes of *Humulus lupulus* have subtelomeric *Kpn*I-repeats (Divashuk et al. 2011).

The orientation of the pseudoautosomal regions on the X chromosome indicates the important role of subtelomeric repeats in sex chromosome genesis. The nature of the Y chromosomes of *Humulus* is puzzling. The unusual sex chromosome system XX/XY1Y2 in *Humulus japonicus* points to the role of chromosome translocations in the karyotype evolution of this species. According to Ohno’s (1967) hypothesis, multiple sex chromosomes have evolved from the standard XX/XY systems by interchanges between autosomes and sex chromosomes. This has been shown in *Silene diclinis* (Lag.) M. Laínz, 1963 (Howell et al. 2009) and may also have occurred in *Humulus japonicus*.
